# Meta-analysis of African ancestry genome-wide association studies identified novel locus and validates multiple loci associated with kidney function

**DOI:** 10.1186/s12864-023-09601-0

**Published:** 2023-08-29

**Authors:** Christopher Kintu, Opeyemi Soremekun, Tafadzwa Machipisa, Richard Mayanja, Robert Kalyesubula, Bernard S. Bagaya, Daudi Jjingo, Tinashe Chikowore, Segun Fatumo

**Affiliations:** 1 The African Computational Genomics (TACG) Research Group, MRC/UVRI and LSHTM Uganda Research Unit, Entebbe, Uganda; 2 Department of Immunology and Molecular Biology, School of Biomedical Sciences, Makerere University College of Health Sciences, Kampala, Uganda; 3 MRC/UVRI and LSHTM Uganda Research Unit, Entebbe, Uganda; 4 Department of Medicine, University of Cape Town & Groote Schuur Hospital, Cape Town, South Africa; 5 African Center of Excellence in Bioinformatics (ACE-B), Makerere University, Kampala, 10101 Uganda; 6 Department of Computer Science, College of Computing, Makerere University, Kampala, Uganda; 7 MRC/Wits Developmental Pathways for Health Research Unit, Department of Paediatrics, Faculty of Health Sciences, University of the Witwatersrand, Johannesburg, South Africa; 8 Sydney Brenner Institute for Molecular Bioscience, Faculty of Health Sciences, University of the Witwatersrand, Johannesburg, South Africa; 9 Channing Division of Network Medicine, Brigham and Women’s Hospital, Boston, MA USA; 10 Harvard Medical School, Boston, MA USA; 11 Department of Non-Communicable Disease Epidemiology, Faculty of Epidemiology and Population Health, London School of Hygiene and Tropical Medicine, London, UK

**Keywords:** GWAS, Africa, Kidney function, Fine mapping, eGFR

## Abstract

Despite recent efforts to increase diversity in genome-wide association studies (GWASs), most loci currently associated with kidney function are still limited to European ancestry due to the underlying sample selection bias in available GWASs. We set out to identify susceptibility loci associated with estimated glomerular filtration rate (eGFRcrea) in 80027 individuals of African-ancestry from the UK Biobank (UKBB), Million Veteran Program (MVP), and Chronic Kidney Disease genetics (CKDGen) consortia.

We identified 8 lead SNPs, 7 of which were previously associated with eGFR in other populations. We identified one novel variant, rs77408001 which is an intronic variant mapped to the *ELN* gene. We validated three previously reported loci at *GATM-SPATA5L1, SLC15A5* and *AGPAT3.* Fine-mapping analysis identified variants rs77121243 and rs201602445 as having a 99.9% posterior probability of being causal. Our results warrant designing bigger studies within individuals of African ancestry to gain new insights into the pathogenesis of Chronic Kidney Disease (CKD), and identify genomic variants unique to this ancestry that may influence renal function and disease.

**Supplementary Information:**

The online version contains supplementary material available at 10.1186/s12864-023-09601-0.

## Introduction

Chronic kidney disease (CKD) is defined as kidney damage or an estimated glomerular filtration rate (eGFR) < 60 ml/min/1.73m^2^ for three or more months [1]. The global prevalence of CKD is estimated to be ~ 10% in adults [2], and sub-Saharan Africa has an estimated prevalence of 13.1% [3]. Care and management of CKD impact a significant financial burden on healthcare systems worldwide, even more so in developing economies, majorly in Africa [4].

The genetic risk factors associated with CKD are ethnically driven including but not limited to telomere length, copy number variation (CNVs), and mitochondrial DNA [5]. Numerous loci related to estimated glomerular filtration rate (eGFR), which is a measure of kidney function for defining CKD, have mainly been discovered using Genome-wide association studies (GWAS) of European and Asian ancestry [6–8]. Owing to the systematic sample selection bias in global GWASs, many of the significant loci discovered in Eurocentric populations can hardly be replicated in African ancestry groups. This is attributed to differences in linkage disequilibrium (LD), differences in heritability, and differences in allele frequencies across ancestries [5, 9–12]. Polymorphisms in two critical genes, the plasma kallikrein gene (*KLKB1*), and the human homolog of the rodent renal failure gene, have been reported to be more associated with End-stage renal disease (ESRD) in the American black population than in whites [13].

In the past 15 years, efforts have been made to increase diversity in cohorts that perform GWASs of complex traits by increasing the number of previously under-represented ancestries, like the African-ancestry [14–17]. However, these efforts are still limited by various logistical challenges. Thus, low power due to the underlying sampling bias towards European ancestry individuals in most global consortia is still eminent [18, 19]. Most such studies have included the African-ancestry population as a small validation cohort in trans-ethnic analyses, mainly for comparison to a much larger European-ancestry cohort. This has not yielded much ancestry-specific discovery of loci to CKD. As a result, such study designs could have missed some of the loci unique to African-ancestry individuals within these global consortia.

Therefore, our study leveraged eGFRcrea GWAS summary data of only African ancestry individuals within three global consortia: Million Veteran Program (MVP), UK Biobank (UKBB), and Chronic Kidney Disease Genetics Consortium (CKDGen consortium), with a combined sample size of over 80 k African-ancestry participants. We aimed to uncover loci associated with CKD in these individuals, further characterize their functionality and phenotype association by performing a Phenome-wide association analysis, and determine the causality of variants using Bayesian Fine-mapping methods.

## Results

### Study samples

GWAS summary statistics of eGFR for 80,027 individuals of African ancestry from two global population-based consortia: UKBB [20] and CKDGen [21], and one Hospital-based cohort, MVP [22] were meta-analyzed. A summary of study sample characteristics is shown in Table 1. Further details of the cohorts contributing to this study are in the Methods section and the Supplementary material.

**Table 1:** Characteristics of the study cohorts

**Study**	**Study full name**	**Ancestry**	**Study design**	**Sample size**	**Transformation**	**References**
UKBB	UK Biobank	African ancestry	Population based	6217	Inverse-variance weighted transformation	Ref [20, 23]
CKDGen consortium	Chronic Kidney Disease Genetics Consortium	African ancestry	Population based	16,474	Log-transformation	Ref [8]
MVP	Million Veteran Program	African ancestry	Hospital based (Out-patient clinics)	57,336	Log-transformation	Ref [24]

### Genome-wide associated SNPs with eGFR

**Fig. 1:**
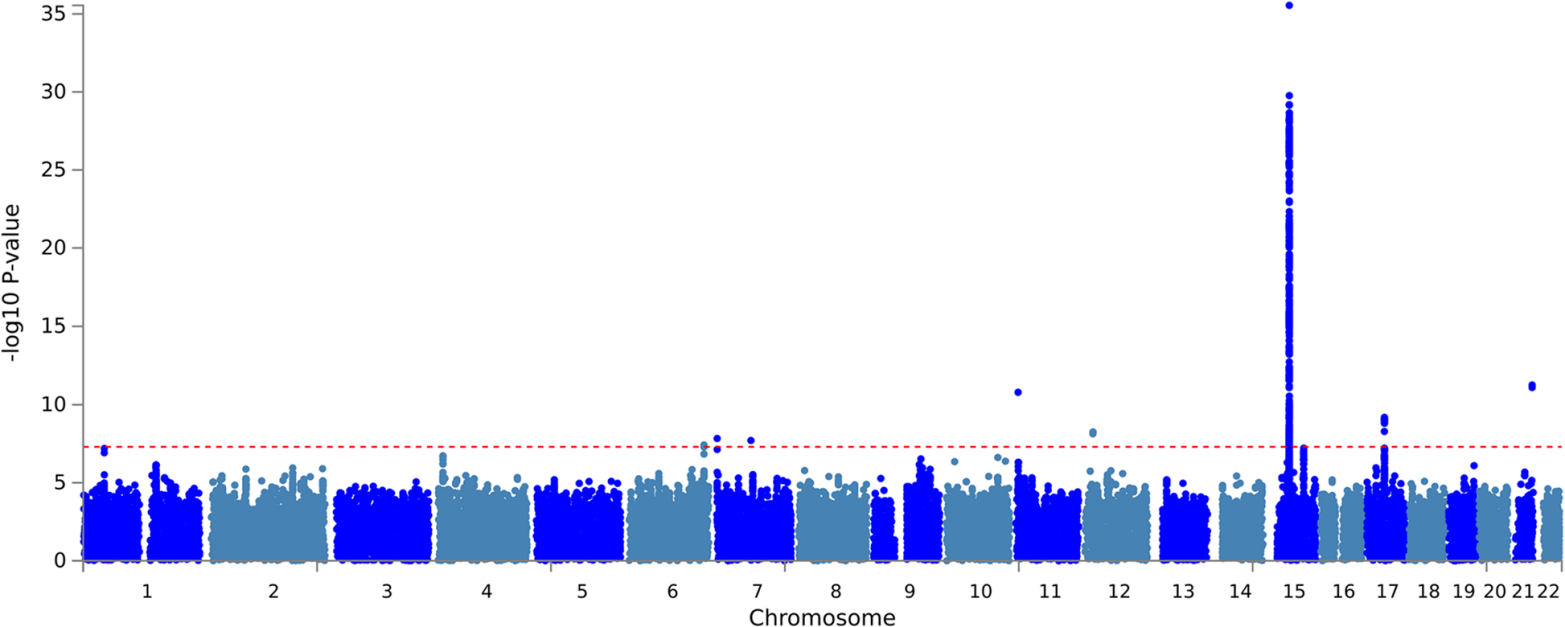
Manhattan plot for Meta-analysis results showing -log10 (*P* value) versus genomic position plots for eGFRcrea. Known lead variants are plotted in red, and novel lead variants are in green

We analyzed ~ 22.6 M SNPs and 403 SNPs from those attained genome-wide significance (*P* < 5 X 10E-8). We identified lead SNPs by clumping at ± 500 kb window around the significant SNPs and identified 8 lead variants. Seven of them had previously been reported in association with eGFRcrea. The smallest and most genome-wide significant *p*-value from the meta-analysis was 1.326e-38 at marker 15:45592887. Figure 1 summarizes the meta-analysis results of African-ancestry eGFRcrea for the three cohorts. Our power calculation indicated an 80% power to determine genome-wide significance in our analyses.

**Fig. 2:**
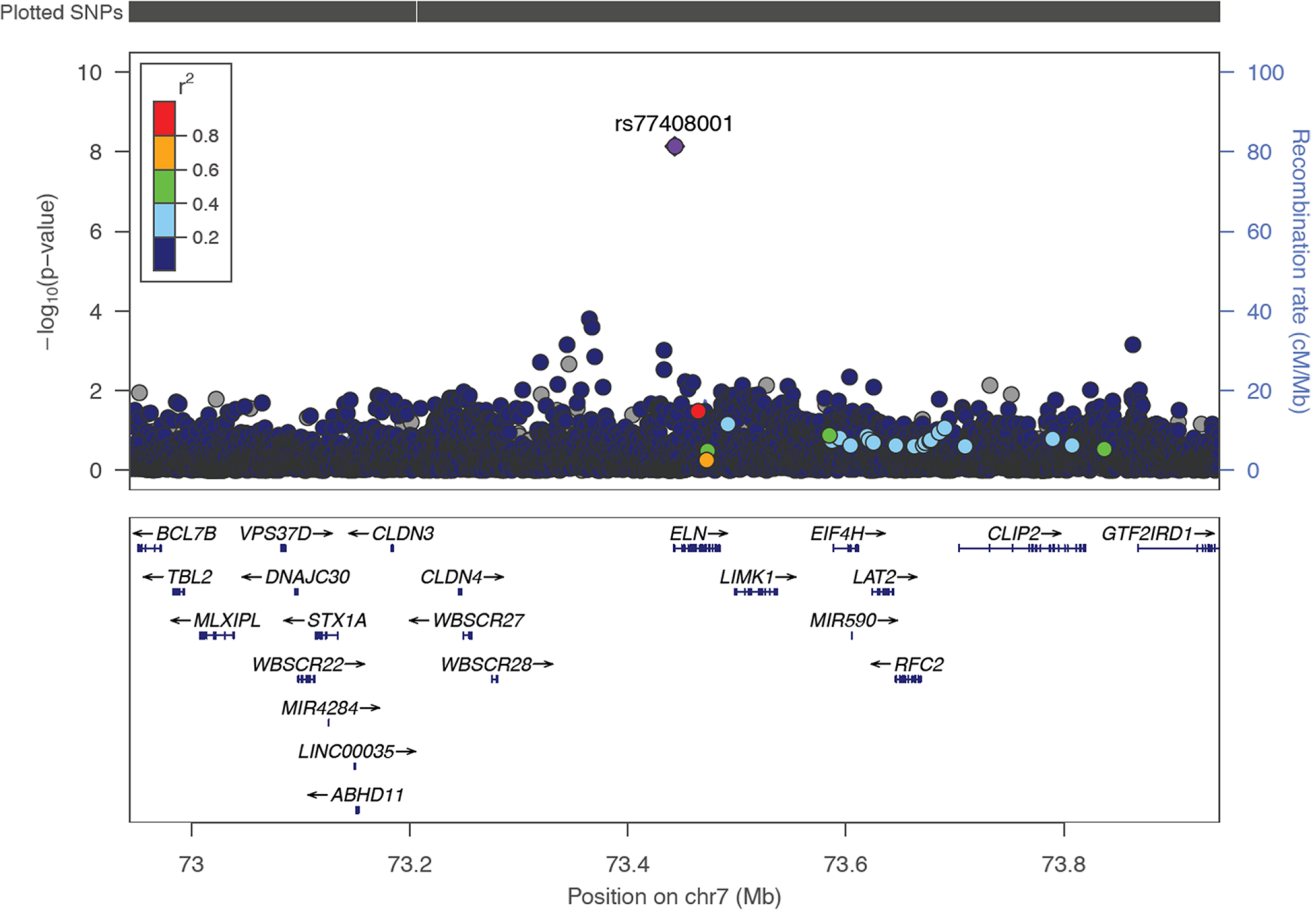
Regional association plot for eGFR in > 80 k African-ancestry individuals from UKBB and MVP consortium at ELN locus. The lead SNP rs77408001 is coloured purple. LD (r2) with other SNPs at the locus was calculated using an African Ancestry reference panel from 1000 Genomes. The arrows represent direction of transcription

SNP rs201602445, located on chromosome 15, at base position 45592887, had the strongest association (*p*-value = 1.326e-38, Fig. 1, Supplementary Table s1). This SNP is located near the spermatogenesis-associated 5-like 1 (*SPATA5L1*) at the GATM-SPATA5L1 locus. We identified one intronic variant, rs77408001, mapping to the *ELN* gene (Table 2, Fig. 2), which had not previously been associated with eGFRcrea, and we considered this as a novel SNP henceforth. This SNP was replicated by proxy in the Uganda genome resource (UGR) dataset [25]. The proxied SNP was rs141339897 (chr7:73727705) (*r* = 1, D’ = 1 in MSL Haplotypes) as shown in Table 2. Other proxied replicated SNPs are shown in Supplementary Table s6, and the locus zoom plot for the most significant proxied SNP (rs3135654) is shown in Supplementary figure s3.

**Table 2:** Genome-wide significant locus (*P* < 5 × 10^–8^) associated with eGFR after meta-analysis

					**UKBB**			**MVP**			**CKDGen**	**Meta-analysis**				**Replication (Uganda)**			
**Locus**	**Lead SNP**	**Chr**	**BP(b37)**	**EA/NEA**	**N**	**MAF**	**P**	**N**	**MAF**	**P**		**P**	**N**	**Zscore**	**Direction**	**N**	**SNP**	**MAF**	**P**
ELN	rs77408001	7	73,443,012	C/A	6217	0.006693	0.03198	57,736	0.0135	7.283e-08	NA	7.264e-09	63,553	-5.210	− −	5877	7:73,727,705	0.01	8.85e-04

*SNP* Single nucleotide polymorphism, *Chr* Chromosome, *BP* Base position, *NEA* Non-effect allele, *SE* Standard error, *NA* Not available

Lead SNP rs10084572 is located near 1-acylglycerol-3-phosphate O-acyltransferase 3 (*AGPAT3*), found on chromosome 21, and lead SNP rs200950799 is located near Solute Carrier Family 15, Member 5 gene (*SLC15A5*) on chromosome 12 have been previously reported in association with eGFRcrea in non-Hispanic blacks. Further information about the genetic architecture of the lead SNPs is illustrated using regional association plots in Supplementary figure s1.

**Fig. 3:**
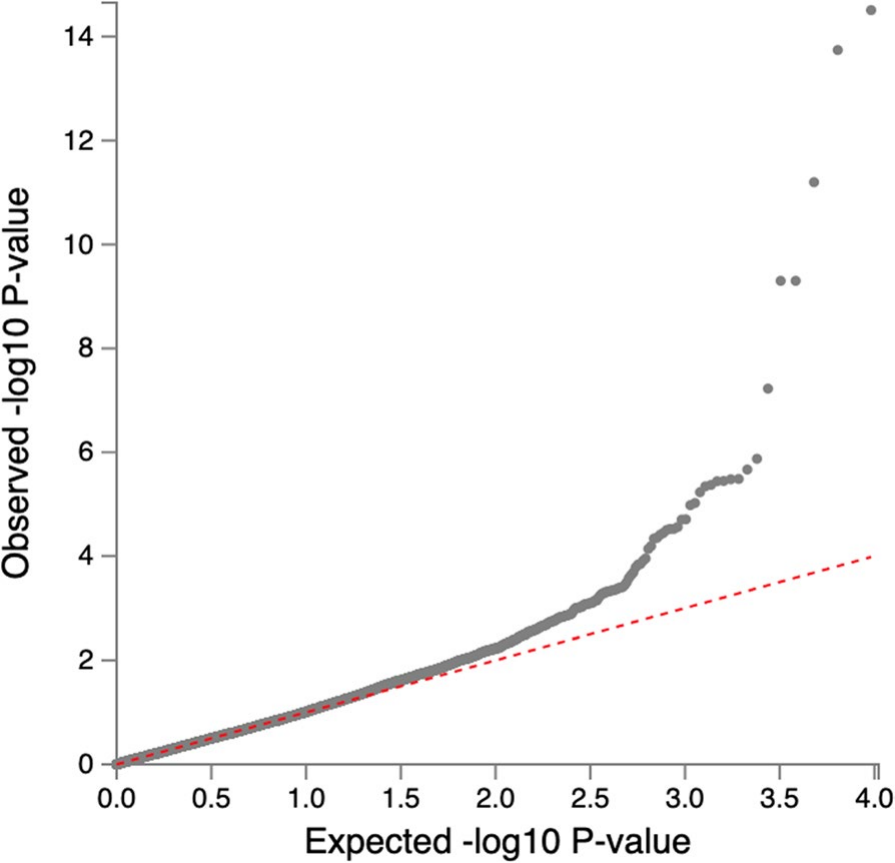
Quantile–Quantile plot of genome-wide associations of eGFR based on serum creatinine

We applied genomic control correction to the meta-analysis results to account for inflation. We found no evidence of significant variation in expected allele frequencies across individuals in each cohort due to residual population structure as shown in Fig. 3. The inflation lambda was 1.07, indicating minimal bias in the test statistics.

### Bayesian fine-mapping

Bayesian fine-mapping was performed based on a ± 500 kb window on each of the lead SNPs from the meta-analysis. Two variants, rs77121243 and rs201602445, each had one credible set accounting for 99% of the posterior probability (PP) of driving the eGFR association in African-ancestry individuals. SNPs 12:17157119 and 21:45412872, each had a credible set-size of 2, accounting for 99% of the PP of being causal SNPs. Variants 7:73443012 (rs77408001), 12:17157119 (rs200950799), and 21:45412872 (rs10084572) had a credible set-size of 5, 2, and 2 respectively (Supplementary Table s2). We then proceeded to compare the 99% credible set sizes of these loci in EUR GWAS. They were smaller 99% credible set sizes in AFR compare to EUR GWAS except for 7:1286192 loci (Supplementary Table s3). This indicated that AFR GWAS enhanced the fine mapping resolution compared to the EUR GWAS.

### eQTL analysis

We conducted kidney expression quantitative trait locus (eQTL) analysis in Genotype-Tissue Expression (GTEx), NephQTL [26], the Human Kidney eQTL Atlas [27], and RegulomeDB [28]. We found significant eQTLs in the GTEx database for 3/8 of the lead SNPs; rs9908131, rs10945657, and rs13230509 (Supplementary table s5) at (*P* < 5E-8). Variants: rs9908131 and rs10945657 had significant eQTLs in both GTEx and NephQTL resources. Variant rs13230509 was not significantly associated with any eQTLs in NephQTLs. For the three variants with significant eQTLs, we concluded that there is evidence of colocalization between eGFR association and eQTLs. All eight lead SNPs were not significant eQTLs in the Human Kidney Atlas. All the lead SNPs showing significant eQTLs were mapped to non-kidney tissues. Using RegulomeDB, we found no evidence of the regulatory impact of the lead SNPs and any of those in high LD.

### SNP association with related phenotypes: PheWAS and MAGMA analysis

Of all the lead SNPs, 7 showed significant association with other phenotypes. SNP variants that showed the most significant association and their phenotypes are shown in supplementary Table s 4. Variants rs77121243 and rs13230509 were most significantly associated with metabolic and haematological traits, respectively. We further performed MAGMA tissue expression analysis, but none of the variants had significant expression in the kidney.

## Discussion

We present the results from a meta-analysis of GWAS summary statistics of eGFR based on serum creatinine, in individuals of African ancestry. To the best of our knowledge, this is one of few studies to combine summary statistics of eGFRcrea from strictly African-ancestry individuals within these 3-major global GWAS consortia (UKBB, CKDGen, and MVP).

None of the lead SNPs identified in our analysis had previously been reported in European ancestry as associated with eGFRcrea. We validated seven variants as mapped to previously reported eGFR loci in individuals of African Ancestry; *GATM/SPATA5L1, SLC15A5*, and *AGPAT3* [7, 8]. The lead SNP with the most significant association is located near the *GATM/SPATA5L1* locus, previously mapped to creatinine synthesis in continental Africans elsewhere [25]. This lead SNP is different from those similarly mapped to the *GATM* locus in individuals of European ancestry [6, 24]. GATM is an enzyme involved in creatinine biosynthesis by encoding glycine amidinotransferase. SNPs at the GATM locus have been reported to have a role in serum creatinine levels without influencing susceptibility to kidney disease in individuals of European ancestry [7, 24]. A similar association has been recently reported in continental Africans [25]. Variant rs13230509 has previously been associated with eGFRcrea in both non-Hispanic blacks and non-Hispanic whites [24].

Locus *SPATA5L1* is a known locus for eGFR [6]. This locus was previously reported to be associated with eGFR in individuals of European ancestry [8]. The mechanism of action of the *SPATA5L1* encoded protein affects kidney function, and how abundant it is in kidney tissues is still not yet clear. Similarly, the *SLC15A5* has previously been associated with eGFR in Europeans [24]. *SLC15A5* encodes a protein that enables symporter activity. It is an integral component of the membrane and is predicted to be involved in peptide transport, protein transport, and transmembrane transport.

One lead SNP, rs77408001, has not yet been mapped to any eGFR loci and is located in the *ELN* gene. This gene encodes a protein, one of two elastic fibre components. The *ELN* gene, also known as the elastin gene, codes for a protein called tropoelastin from which Elastin is synthesized. Elastin is the major component of elastic fibres, which support the body's connective tissues [29]. A deletion mutation in this gene at 7q11.23 has been linked to Williams syndrome (WS), characterized by cardiovascular disease including elastin arteriopathy, peripheral pulmonary stenosis, supravalvar aortic stenosis, and hypertension [30].

The bayesian fine-mapping analysis identified two lead SNPs, rs77121243 and rs201602445, with a single credible set-size each showing a 99.9% posterior probability of driving the association at respective loci. The lead SNP; rs201602445, located near the more characterized locus; *GATM*, is significantly associated with serum creatinine in the African-ancestry population [25]. Overall, the aggregation of African Ancestry GWAS enhanced the fine mapping resolution compared to European GWAS.

Most of the lead variants were not significant expression quantitative trait loci (eQTLs) in publicly available kidney gene expression resources, and they did not co-localize to any kidney tissues. This is unsurprising considering the lack of generalizability across populations in these resources. For example, over 80% of the expression quantitative trait locus (eQTL) is from individuals of European ancestry [31]. The most significant PheWAS association was with eGFR, and two SNPs, rs10084572 and rs77408001, were associated with Height and Total cholesterol in large VLDL, respectively, but these were not significant associations.

Our study had some limitations, for example, diabetes and hypertension are common risk factors for kidney damage [32], however, we were not able to perform a stratified analysis based on underlying patient kidney disease comorbidities like diabetes and hypertension within these cohorts. This would enable us to identify which comorbidity groups contributed the most toward observed associations. Our largest contributing cohort, MVP, had previously reported diabetes-stratified GWAS of over 9 k individuals with 32 genome-wide significant variants [24]. They reported that one of the loci, SPATA5L1, identified in this study had the strongest association in the non-diabetics group. In this study, we only had access to GWAS summary statistics from these cohorts with no socio-demographics information, and thus couldn’t conduct this analysis.

Another potential limitation to our findings is the large effect size and standard error of the novel SNP rs77408001 in both the meta-analysis and the MVP cohort. We mainly attributed this to different phenotype transformations by individual studies. For example, the UKBB is Inverse-rank normal transformation, the MVP dataset has no transformation, and the CKDGen is log transformed. It is for this particular reason that we performed a *p*-value based meta-analysis.

However, another potential limitation is that the Imputation quality information for the SNP, rs77408001, associated with the novel loci, was not available in the summary statistics of either study. This, in addition to our inability to conduct a direct replication analysis for this SNP, makes it difficult to fully affirm the true genetic effect of this SNP on eGFR in African ancestry individuals. We recommend future similarly designed studies to critically characterize this variant in more detail.

Overall, our findings uncover a potential new locus for eGFR in African-ancestry individuals and further affirm those previously reported in other ancestries. Some of the variants reported here are unique to the African ancestry, and therefore this warrants larger ancestry-specific meta-analyses to decipher more loci associated with CKD in Africans. Furthermore, we justify here that even African-ancestry individuals outside Africa have genetic differences from continental Africans, and caution should be taken not to generalize individuals within this ancestry while conducting GWASs.

## Methods

### Study cohort characteristics

#### Million Veteran Program (MVP)

A detailed description of the MVP cohort has been described elsewhere [24]. Briefly, the MVP cohort recruited fully consented participants from 63 Veteran American (VA) medical facilities. Out of the several phenotypes recorded, 280722 participants have eGFR analysis available. Of these, 57336 are individuals of African ancestry. Most participants with eGFR analysis are non-diabetics, with just ~ 33% being diabetics. The participants are further stratified into hypertensives and non-hypertensives. About 93% of hypertensive diabetics are non-Hispanic blacks, but eGFR is generally higher in non-Hispanic blacks than non-Hispanic whites in this cohort. We included all individuals (*n* = 57336) of African ancestry with eGFR analyzed from the MVP cohort, and we didn’t stratify them based on any underlying clinical diagnoses. More information about this cohort can be found in the Supplementary methods.

#### Chronic Kidney Disease Genetics (CKDGen) consortium

This is a collaborative consortium of mainly population-based studies from different ethnicities. The consortium follows a centralized analysis plan where each participating study performs an independent analysis following a centralized analysis plan. Depending on the age of participants, the studies contributing to the CKDGen consortium generally estimate the eGFR based on serum creatinine using four-variable Modification of Diet in Renal Disease Study Equation [8] or the Schwartz equations [33]. The effect of outliers on eGFR values is harmonized by averaging at 15 and 200 ml/min/1.73 m2. Further information about the specific study within the consortium, from which participants included in our study were obtained can be found at https://doi.org/10.1038/ncomms10023 [8]. This study recruited 16474 participants of African ancestry, with information on eGFRcrea analysis. More details about the CKDGen consortium can be found in Supplementary methods.

#### UK BioBank (UKBB)

The UKBB is a large prospective cohort with > 50 k participants aged 40–73 years. The details of steps followed during enrolment have been previously described elsewhere [20, 23]. At baseline, each participant completed a detailed, computerized questionnaire. Biochemical tests and genotyping were conducted on archived blood specimen. For longitudinal follow-up, study data was linked to participant’s national health records. Phenotype expansion is still on-going. Estimation of glomerular filtration rate based on serum creatinine was calculated using the CKD-EPI equation [34]. From the UKBB cohort, our study included 6217 individuals of African ancestry with analyzed eGFRcrea. The supplementary methods have further details on this cohort. Ethnicity was coded as white, black, south Asian or other based on survey questionnaires. However, for purposes of eGFR calculation, ethnicity was coded as black or other.

#### Meta-analysis

We aggregated summary statistics by performing the Stouffer’s method implemented in METAL due to the different GWAS trait transformations that had been applied by the individual studies [35]. Two of the datasets (UKBB and MVP) had their genome coordinates in build GRCh37/hg19. The genome coordinates for the CKDGen dataset used in this analysis were reported in b36 but were converted to build GRCh37/hg19 before the meta-analysis. Briefly, this method converted the two-sided *p*-value to a z-statistic which reflected the direction of association based on the reference allele. Each z-score was then weighted; the squared weights were chosen to sum to 1, and each cohort-specific weight was proportional to the square root of the adequate number of individuals in that cohort. The weighted z-statistics were then summed across the three cohorts, and the summary z-score was converted to a two-sided *p*-value. We performed double GC correction once during the study-specific analyses and repeated the statistics from the meta-analysis. QQ-plots for the individual datasets and the meta-analysis are presented in the Supplementary data.

#### Fine-mapping

We used a Bayesian method to fine-map all SNPs within 500 kb of the eight lead SNPs [36]. This approach has been detailed elsewhere [36]. Briefly, we used the Z-score for the ith SNP (Zi) to calculate the Baye’s factor in favour of the association, denoted BFi, using equation (i) below:

1 $${BF}_{i}={e}^{\left[\frac{{Z}_{1}^{2}-\mathrm{log}(\mathrm{K})}{2}\right]},$$

where K is the number of studies.

The posterior probability (PP) for the ith SNP was calculated using equation (ii) below;

2 $${\pi }_{i}=\frac{{\mathrm{BF}}_{\mathrm{i}}}{{\Sigma }_{j}{\mathrm{BF}}_{\mathrm{j}}}$$

where $\mathrm{\Sigma BFi}$ denotes the total sum of SNPs at that locus. We obtained the 99% credible sets using Bayes’ factors of the SNPs after rearranging them in descending order. We only counted those SNPs that had a posterior probability of ≥ 0.99.

### Exploring functional mapping and annotation

#### FUMA analysis

To perform gene annotation and functional mapping for each lead SNP, we used a web-based platform called FUMA. This resource uses information from multiple biological resources to facilitate functional annotation of GWAS results, gene prioritization, and interactive visualization [37].

#### PheWAS analysis

We characterized the functionality of the lead SNPs using the global GWAS ATLAS resource [38]. This resource compiled a catalogue of > 4 k GWAS results across over 3000 unique traits in 28 domains. Leveraging this resource, for each lead SNP, we assessed the genetic nature of these variants in effect size distribution, minor allele frequency, and biological functionality of the traits associated with the lead variants. We also characterized the extent of pleiotropy at a trait-associated locus and associated genes and investigated trait polygenicity and SNP heritability.

#### MAGMA gene set analysis

Gene set analysis based on *P*-values was performed using MAGMA [39], a tool embroiled within FUMA. The method performs gene-based *P*-value analysis for protein-coding genes by mapping SNPs to genes if they are located within the same genes. The default MAGMA setting used an SNP-wise model for gene analysis and a competitive model for gene set analysis. Multiple testing was corrected using the Bonferroni correction for gene analysis and FDR for gene-set research.

#### eQTL analysis

We leveraged four publicly available kidney eQTL resources: GTEx [31], NephQTL [26], the Human Kidney eQTL Atlas, and RegulomeDB to perform expression quantitative trait loci analysis. For each lead SNP, we searched eQTLs associated with each lead SNP in the kidney cortex, glomerular and tubulointerstitial kidney tissue, whole kidney, glomerulus and tubules in GTEx, NephQTL, and Human kidney eQTL Atlas, respectively. We used RegulomeDB to determine the regulatory impact of each lead SNP.

#### Power analysis

The power to achieve genome-wide significance at *p* < 5 × 10^–8^ was estimated with the software MetaGAP [40]. The link to the tool can be found at http://www.devlaming.eu/metagap.html. For the genetic association analyses, a meta-analysis of ~ 80,000 individuals will achieve 80% power to detect association of a common genetic variant that explains 0.05% of the variance in eGFR at a stringent genome-wide significance level (*p* < 5 × 10–8).

### Supplementary Information

**Additional file 1:** **Supplementary methods.** **Table S1.** Association results for meta-analysis lead SNPs. **Table S2.** Annotation-informed fine-mapping using the AFR meta-analyzed dataset. **Table S3.** Annotation-informed fine-mapping using the AFR and EUR datasets. **Table S4.** PheWAS association analysis. **Supplementary Table s5.** Colocalization with gene expression from GTEx and NephQTL. **Table S6.** Proxy SNPs with some evidence of association with lead SNP rs77408001 (+/- 500kb) within the CKDGen dataset. **Figure S1.** Regional association plots showing Genetic architecture of the genome-wide significant susceptibility variants for CKD (a)-(h). The most significant SNP in each region is plotted in blue. LD based on the 1000G sample is color-coded red (r2 to top SNP 0.8–1.0), orange (0.5–0.8), yellow (0.2–0.5) and blue (<0.2). **Figure S2.** The Phenome-wide association (PheWAS) plot shows the significant (*p* ≤ 0.05) associations of 7:73443012:C:A / rs77408001 for all available traits, generated by bottom-line integrative analysis across all datasets in the Portal. The triangle data points indicate direction of effect. **Supplementary figure s3.** Regional association plot for proxy SNP rs3135654 in 16474 African-ancestry individuals from the CKDGen consortium dataset at the ELN locus.

## Data Availability

The MVP dataset was accessed upon request using the accession number dbgap proposal #30287. The UKBB eGFRcrea summary statistics dataset was accessed from the UK biobank using the phenocode 30700. The CKDGen dataset was accessed on the CKDGen consortium (https://ckdgen.imbi.uni-freiburg.de/). All other materials and related data in this study are available upon request directed to segun.fatumo@lshtm.ac.uk.

## References

[1] Eknoyan G, Lameire N, Eckardt K, Kasiske B, Wheeler D, Levin A, Stevens PE, Bilous RW, Lamb EJ, Coresh JJ. KDIGO 2012 clinical practice guideline for the evaluation and management of chronic kidney disease. Kidney int. 2013;3(1):5–14.

[2] Eckardt K-U (2013). Evolving importance of kidney disease: from subspecialty to global health burden. Lancet.

[3] Ajayi SO (2021). Prevalence of chronic kidney disease as a marker of hypertension target organ damage in Africa: a systematic review and meta-analysis. Int J Hypertens.

[4] Xie Y (2018). Analysis of the Global Burden of Disease study highlights the global, regional, and national trends of chronic kidney disease epidemiology from 1990 to 2016. Kidney Int.

[5] Canadas-Garre M (2019). Genetic susceptibility to chronic kidney disease - some more pieces for the heritability puzzle. Front Genet.

[6] Wuttke M (2019). A catalog of genetic loci associated with kidney function from analyses of a million individuals. Nat Genet.

[7] Kottgen A (2009). Multiple loci associated with indices of renal function and chronic kidney disease. Nat Genet.

[8] Pattaro C (2016). Genetic associations at 53 loci highlight cell types and biological pathways relevant for kidney function. Nat Commun.

[9] Fatumo S (2020). The opportunity in African genome resource for precision medicine. EBioMedicine.

[10] Deepti Gurdasani TC, Tekola-Ayele Fasil, Pagani Luca, Tachmazidou Ioanna (2015). The African Genome Variation Project shapes medical genetics in Africa. Nature.

[11] Gurdasani D (2019). Uganda Genome Resource Enables Insights into Population History and Genomic Discovery in Africa. Cell.

[12] Udler MS (2015). Effect of Genetic African Ancestry on eGFR and Kidney Disease. J Am Soc Nephrol.

[13] Hsu CY (2003). Racial differences in the progression from chronic renal insufficiency to end-stage renal disease in the United States. J Am Soc Nephrol.

[14] Barroso I (2021). The importance of increasing population diversity in genetic studies of type 2 diabetes and related glycaemic traits. Diabetologia.

[15] Kelleher, J., et al., 2018.

[16] Mulder N (2018). H3Africa: current perspectives. Pharmgenomics Pers Med.

[17] Ramirez AH (2022). The All of Us Research Program: Data quality, utility, and diversity. Patterns (N Y).

[18] Improving equity in human genomics research. Commun Biol, 2022. 5(1): 281. 10.1038/s42003-022-03236-9 PMC8948221 35332248

[19] Peterson RE (2019). Genome-wide association studies in ancestrally diverse populations: opportunities, methods, pitfalls, and recommendations. Cell.

[20] Allen N (2012). UK Biobank: current status and what it means for epidemiology. Health Policy Technol.

[21] Kottgen A, Pattaro C (2020). The CKDGen Consortium: ten years of insights into the genetic basis of kidney function. Kidney Int.

[22] Gaziano JM (2016). Million Veteran Program: a mega-biobank to study genetic influences on health and disease. J Clin Epidemiol.

[23] Cathie Sudlow, J.G., Naomi Allen, Valerie Beral, Paul Burton, JohnDanesh,PaulDowney,PaulElliott, Jane Green, Martin Landray, Bette Liu, Paul Matthews, Giok Ong,Jill Pell, Alan Silman, Alan Young, Tim Sprosen, TimPeakman,RoryCollins, UKBiobank: An Open Access Resource for Identifying the Causes of a Wide Range of Complex Diseases of Middle and Old Age. 2015. 10.1371/journal.pmed.1001779 PMC4380465 25826379

[24] Hellwege JN (2019). Mapping eGFR loci to the renal transcriptome and phenome in the VA Million Veteran Program. Nat Commun.

[25] Fatumo S (2021). Discovery and fine-mapping of kidney function loci in first genome-wide association study in Africans. Hum Mol Genet.

[26] Gillies CE (2018). An eQTL Landscape of Kidney Tissue in Human Nephrotic Syndrome. Am J Hum Genet.

[27] Sheng X (2021). Mapping the genetic architecture of human traits to cell types in the kidney identifies mechanisms of disease and potential treatments. Nat Genet.

[28] Dong, S., et al., 2022.

[29] Brooke B (2003). New insights into elastin and vascular disease. Trends Cardiovasc Med.

[30] Schubert C (2009). The genomic basis of the Williams-Beuren syndrome. Cell Mol Life Sci.

[31] Consortium, G.T. (2013). The Genotype-Tissue Expression (GTEx) project. Nat Genet.

[32] Kazancioglu R (2013). Risk factors for chronic kidney disease: an update. Kidney Int Suppl (2011).

[33] Schwartz GJ (2012). Improved equations estimating GFR in children with chronic kidney disease using an immunonephelometric determination of cystatin C. Kidney Int.

[34] Andrew S. Levey, L.A.S., Christopher H. Schmid, Yaping (Lucy) Zhang, Alejandro F. Castro, Harold I. Feldman, John W. Kusek, Paul Eggers, Frederick Van Lente, Tom Greene, and Josef Coresh, A New Equation to Estimate Glomerular Filtration Rate*.* 2009. 10.7326/0003-4819-150-9-200905050-00006 PMC2763564 19414839

[35] Willer CJ, Li Y, Abecasis GR (2010). METAL: fast and efficient meta-analysis of genomewide association scans. Bioinformatics.

[36] Wellcome Trust Case Control, C (2012). Bayesian refinement of association signals for 14 loci in 3 common diseases. Nat Genet.

[37] Watanabe K (2017). Functional mapping and annotation of genetic associations with FUMA. Nat Commun.

[38] Watanabe K (2019). A global overview of pleiotropy and genetic architecture in complex traits. Nat Genet.

[39] de Leeuw CA (2015). MAGMA: generalized gene-set analysis of GWAS data. PLoS Comput Biol.

[40] de Vlaming R (2017). Meta-GWAS Accuracy and Power (MetaGAP) calculator shows that hiding heritability is partially due to imperfect genetic correlations across studies. PLoS Genet.

