# Supplementary material for: Meta-analysis of African ancestry genome-wide association studies identified novel locus and validates multiple loci associated with kidney function
Source: BMC Genomics. 2023 Aug 29;24:496. doi: 10.1186/s12864-023-09601-0 (PMC10464349; doi:10.1186/s12864-023-09601-0)
Supplement: Supplementary file 1 — Additional file 1: Supplementary methods. Table S1. Association results for meta-analysis lead SNPs. Table S2. Annotation-informed fine-mapping using the AFR meta-analyzed dataset. Table S3. Annotation-informed fine-mapping using the AFR and EUR datasets. Table S4. PheWAS association analysis. Supplementary Table s5. Colocalization with gene expression from GTEx and NephQTL. Table S6. Proxy SNPs with some evidence of association with lead SNP rs77408001 (+/- 500kb) within the CKDGen dataset. Figure S1. Regional association plots showing Genetic architecture of the genome-wide significant susceptibility variants for CKD (a)-(h). The most significant SNP in each region is plotted in blue. LD based on the 1000G sample is color-coded red (r2 to top SNP 0.8–1.0), orange (0.5–0.8), yellow (0.2–0.5) and blue (<0.2). Figure S2. The Phenome-wide association (PheWAS) plot shows the significant (p ≤ 0.05) associations of 7:73443012:C:A / rs77408001 for all available traits, generated by bottom-line integrative analysis across all datasets in the Portal. The triangle data points indicate direction of effect. Supplementary figure s3. Regional association plot for proxy SNP rs3135654 in 16474 African-ancestry individuals from the CKDGen consortium dataset at the ELN locus. [file 12864_2023_9601_MOESM1_ESM.docx]

**Supplementary methods**

**Study cohort characteristics**

**Million Veteran Program (MVP).**

The MVP is a USA-based multi-ethnic cohort with over 930,000 veterans recruited from 63 VA medical facilities. Baseline surveys and lifestyle characteristics surveys have been conducted in-person since 2011. It is from these surveys that information on race and ethnicity is obtained. This program was approved by the VA Central Institutional Review Board. Upon provision of informed consent, participants grant access to their medical records, a blood sample, and permission to follow-up. Genotyping was performed on the Affymetrix Axiom Biobank Array chip included coverage of African and Hispanic haplotypes. Samples are anonymized for downstream research purposes to make it impossible to link study data to participants.

Phlebotomists collected a blood sample from each consented participant and shipped it to a central biorepository in Boston, Massachusetts for biobanking. DNA extraction and genotyping was performed at two sites. The MVP genomics working group then applied using standardized quality control (QC) and genotype calling algorithms using the Affymetrix Power Tools Suite (v1.18). Some of the QC parameters included removal of duplicate samples, checking for sex inconsistency between reported and genotyped sex, and checking for heterozygosity. Relatedness was checked using the KING software [1].

Variants were excluded based on deviations from expected allele frequency, and other variant-specific parameters, within the 1000 Genomes project before imputing. Pre-phasing was performed using EAGLE v282, and then imputation was performed Minimac3 software [2]. FlashPCA was used for Principal component analysis (PCA) and the top ten principal components explained the greatest variability.

Race and ethnicity were based on a self-report method using standardized survey forms. If these were un-available, observational medical outcomes partnership (OMOP) data were used. Race and ethnicity were categorized as whites and non-Hispanic blacks.

Glomerualr filtration rate (GFR) at baseline was obtained using serum creatinine closest to enrolment, and creatinine was measured using the IDMS reference method. The eGFR CKD-EPI equation was used to calculate GFR. Exclusion criteria was individuals on dialysis, amputees, kidney transplant individuals, and those on HIV medications which could increase creatinine clearance.

MVP GWAS analysis was performed using performed linear regression association tests with additive models for untransformed eGFR. The covariates adjusted for included age, at eGFR measure, age2, sex, BMI, and the top ten genetic principal components (PCs) in analyses. All regression- based analyses were conducted in SNPTEST-v2.5.4-beta86. Inference was limited to genotyped and imputed variants with SNPTEST Info scores of 0.4 or higher, with Hardy-Weinberg equilibrium p-value > 5 × 10−8 for common variant analysis (MAF > 0.1). Meta-analyses across race and strata were performed using fixed- effects, inverse variance-weighted meta-analysis implemented in METAL [3]. The genome coordinates for the MVP dataset were in build GRCh37/hg19. Further MVP cohort analyses relevant to the eGFRcrea phenotype have been fully described by Jacklyn N. Hellwege *et al*., elsewhere [4].

**Chronic Kidney Disease Genetics (CKDGen) Consortium**

The Chronic Kidney Disease Genetics (CKDGen) Consortium is a collaborative effort of studies from different ethnicities that perform GWASs of renal function traits with a central objective to understand the genetic basis of Chronic Kidney Disease (CKD). GWASs contributing studies are meta-analyzed following a centralized analytical plan. The consortium has over 700k individuals overall, with majority (~ 74%) being of European ancestry, East Asians are ~ 29%, and African ncestry individuals contribute the least, ~ 0.03%.

Most participating studies use the CKD-EPI equation to calculate eGFRcrea and the MDRD equation. This is a continuously updated consortium and the most recent median age for all studies is 54 years, and the median mean eGFR is 89 ml/min/1.73 m2 (interquartile range: 81–94). Imputation for individual studies was performed based on the Haplotype Reference Consortium (HRC) v1.1 or the 1000 Genomes Project phase 3 v5 ALL or phase 1 v3 ALL panels. Only variants with imputation quality r2 ≥ 0.3 were included in the analyses. Sex- and age-adjusted linear regression models were fitted to the logarithm of eGFR.

GWAS were performed based on additive genetic effects, by regressing the residuals of the linear models on SNP dosage levels. Relatedness was accounted for using family-based studies, including genetic principal component analysis. SNPs were selected based on imputation quality score >0.6, and minor allele count >10. SNPs not present in at least half the studies meta-analyzed are discarded form further analysis. Further details about this cohort can be found at <https://ckdgen.imbi.uni-freiburg.de/> and the particular study characteristics included in our analysis have been documented elsewhere by Pattaro *et al*., [5].

**UK Biobank (UKBB)**

The UK Biobank (UKBB) is a UK-based study with 502,536 individuals of age 40 to 73 years recruited between 2007-2010. These were recruited from 22 assessment centers in the UK. Each consented participant provided biological data and information using questionnaires that were collected at baseline. The details of the baseline surveys have been reported elsewhere [6]. Ethnicity was initially coded as white, black, south Asian or other, but this was changed to black or other during eGFR calculations.

Individuals with prevalent end-stage kidney disease (ESKD), those receiving renal replacement therapy at baseline, those with eGFR measure of <15 ml/min/1.73m^2^, and those with self-reported ESKD were excluded from the analyses.

Those with prevalent ESKD or who were receiving renal replacement therapy in any form at baseline were excluded, defined from self-reported ESKD according to a pre-specified algorithm. Any participant with a calculated eGFR from any measure of <15 ml/min/1.73m^2^ was excluded. Participants with previous history of CVD (self-reported angina, myocardial infarction, stroke or transient ischaemic attack) were excluded.

Details of down-stream sampling, handling, and quality control of the blood samples has been described elsewhere [7, 8]. Briefly, Serum and urine creatinine were measured using an enzymatic (creatinase), IDMS-traceable, method on Beckman Coulter AU5400 instrument [8]. Variables were adjusted for after data were centrally adjusted by the UKBB team. The ancestries in UKBB are self-reported. Imputation was performed based on the UK10K haplotype reference panels. The first 20 PC’s were included in the covariates. The genome coordinates for the UKBB dataset were in build GRCh37/hg19.

**Replication cohort: Uganda Genome Resource**

We attempted replication in the Uganda Genome Resource dataset [9, 10]. The Uganda genome resource (UGR) is a population-based cohort, code-named general population cohort (GPC), that started recruiting participants in the 1980’s. The recruitment was conducted by Medical Research Council (MRC) of the United Kingdom (UK) in partnership with the Uganda Virus Research Institute (UVRI) initially as an HIV-cohort. The study recruited approximately 22000 participants from 25 villages in the South-western district of Uganda called Kyamulibwa.

With increasing prevalence of Non-communicable diseases, the recruitment criteria of this longitudinal cohort were expanded to investigate the genetics and epidemiology of communicable and non-communicable diseases. This was to provide evidence-based insights by leveraging the GPC round 22 that was initiated in 2011 through a collaborative effort by the University of Cambridge, Wellcome Sanger Institute (WSI), and MRC/UVRI. There are approximately 7000 individuals with genotyped data in this cohort using the HumanOmni2.5-8 Illumina genotyping chip array. Further information about this cohort has been detailed elsewhere [9, 10].

**Supplementary tables and figures**

**Table S1: Association results for meta-analysis lead SNPs**

| **SNP** | **rsID** | **Nearest gene** | **EA** | **NEA** | **MAF** | **P-value** |
| --- | --- | --- | --- | --- | --- | --- |
| 6:160652929 | rs10945657 | *SLC22A2* | A | G | 0.0955 | 1.747e-08 |
| 7:1286192 | rs13230509 | *UNCX* | G | C | 0.0268 | 4.631e-09 |
| 7:73443012 | rs77408001 | *ELN* | A | C | 0.000 | 7.264e-09 |
| 12:17157119 | rs200950799 | *SLC15A5* | C | T | 0.0021 | 1.709e-09 |
| 15:45592887 | rs201602445 | *CTD* | A | T | 0.0005 | 1.326e-38 |
| 17:37520449 | rs7208487 | *FBXL20* | G | T | 0.311 | 2.277e-10 |
| 21:45412872 | rs10084572 | *AGPAT3* | T | C | 0.98 | 1.067e-12 |
| 11:5248232 | rs77121243 | *HBB* | T | A | 0.0030 | 2.74e-14 |

**Table S2: Annotation-informed fine-mapping using the AFR meta-analyzed dataset**

| **SNP** | **rsID** | **CHR** | **BP** | **Zscore** | **BF** | **postprob** | **ppcumsum** | **credible set size** |
| --- | --- | --- | --- | --- | --- | --- | --- | --- |
| 6:160668389 | rs10945657 | 6 | 160668389 | 4.778 | 64091.5885045479 | 0.011140701767357 | 0.931622546512688 | 12 |
| 7:1286192 | rs13230509 | 7 | 1286192 | -5.666 | 6617342.48311767 | 0.813425357508344 | 0.813425357508344 | 5 |
| 7:73443012 | rs77408001 | 7 | 1285195 | 4.74 | 53488.7381961992 | 0.00657501045184151 | 0.989430080865739 | 5 |
| 11:5248232 | rs77121243 | 11 | 5248232 | -6.738 | 5106338303.89765 | 0.999907011523534 | 0.999907011523534 | 1 |
| 12:17157119 | rs200950799 | 12 | 17157119 | -5.83 | 16985598.734235 | 0.563191851140422 | 0.563191851140422 | 2 |
| 15:45592887 | rs201602445 | 15 | 45592887 | NA | NA | NA | NA | 263130 |
| 17:37543449 | rs7208487 | 17 | 37543449 | -6.142 | 109946936.258997 | 0.182374491955851 | 0.41002741940495 | 11 |
| 21:45412872 | rs10084572 | 21 | 45412872 | -6.893 | 14685859216.922 | 0.589989777993271 | 0.589989777993271 | 2 |

**Table S3: Annotation-informed fine-mapping using the AFR and EUR datasets**

| **SNP** | **CHR** | **BP** | **EA** | **NEA** | **MAF(AFR)** | **99% credible set size (AFR)** | **99%credible set size (EUR)** |
| --- | --- | --- | --- | --- | --- | --- | --- |
| 6:160668389 | 6 | 160668389 | A | G | 0.0210155 | 12 | 21 |
| 7:1286192 | 7 | 1286192 | G | C | 0.5606187 | 4 | 3 |
| 7:73443012 | 7 | 1285195 | C | A | 0.0228600 | 5 | 2182 |
| 11:5248232 | 11 | 5248232 | T | A | 0.0000064 | 1 | 53 |
| 12:17157119 | 12 | 17157119 | C | T | 0.0190310 | 2 | 2339 |
| 15:45592887 | 15 | 45592887 | A | T | 0.0415119 | 1 | 18 |
| 17:37543449 | 17 | 37543449 | T | G | 0.1846994 | 11 | 181 |
| 21:45412872 | 21 | 45412872 | T | C | 0.4997346 | 2 | 3001 |

**Table S4: PheWAS association analysis**

| **rsID** | **SNP** | **chr** | **bp** | **EA** | **NEA** | **Trait** | **P-value** |
| --- | --- | --- | --- | --- | --- | --- | --- |
| rs77121243 | 11:5248232 | 11 | 5248232 | T | A | Estimated glomerular filtration rate | 0.002677888 |
| rs201602445 | 15:45592887 | 15 | 45592887 | A | T | Estimated glomerular filtration rate | 4.02243E-05 |
| rs7208487 | 17:37543449 | 17 | 37543449 | G | T | Estimated glomerular filtration rate | 1.50E-12 |
| rs10084572 | 21:45412872 | 21 | 45412872 | T | C | Height | 0.02348512 |
| rs2279463 | 6:160668389 | 6 | 160668389 | A | G | Estimated glomerular filtration rate | 2.90E-16 |
| rs13230509 | 7:1286192 | 7 | 1286192 | G | C | Estimated glomerular filtration rate | 3.04E-08 |
| rs77408001 | 7:73443012 | 7 | 73443012 | A | C | Total cholesterol in large VLDL | 0.049793 |

**Supplementary Table s5: Colocalization with gene expression from GTEx and NephQTL**

|  |  |  |  |  |  | **GTEX** |  |  |  | **NephQTL** |  |
| --- | --- | --- | --- | --- | --- | --- | --- | --- | --- | --- | --- |
| **rsID** | **SNP** | **chr** | **bp** |  | **pvalue** | **Normalized Effect Size** | **Tissue** |  | **Beta** | **t-statistic** | **pvalue** |
| rs77121243 | 11:5248232 | 11 | 5248232 |  | NA | NA | NA |  | NA | NA | NA |
| rs200950799 | 12:17157119 | 12 | 17157119 |  | NA | NA | NA |  | NA | NA | NA |
| rs201602445 | 15:45592887 | 15 | 45592887 |  | NA | NA | NA |  | NA | NA | NA |
| rs7208487 | 17:37543449 | 17 | 37543449 |  | 2.50E-12 | -0.34 | Esophagus-Mucosa |  | 0.24 | 2.2 | 0.033 |
| rs10084572 | 21:45412872 | 21 | 45412872 |  | No significant eQTLs were found | | |  | -0.37 | -1.6 | 0.11 |
| rs2279463 | 6:160668389 | 6 | 160668389 |  | 7.10E-10 | 0.43 | Skin- Not Sun Exposed (Suprapubic) |  | -0.23 | -2.4 | 0.018 |
| rs13230509 | 7:1286192 | 7 | 1286192 |  | 1.20E-11 | 0.39 | Testis |  | 0.15 | 1.3 | 0.2 |
| rs77408001 | 7:73443012 | 7 | 73443012 |  | No significant eQTLs were found | | |  | NA | NA | NA |

**Table S6: Proxy SNPs with some evidence of association with lead SNP rs77408001 (+/- 500kb) within the CKDGen dataset**

| **rsID** | **Chr** | **bpos** | **A1** | **A2** | **FRQ** | **Beta** | **SE** | **P** | **D’** |
| --- | --- | --- | --- | --- | --- | --- | --- | --- | --- |
| rs3135654 | 7 | 73668312 | A | G | 0.738 | -0.028 | 0.0076 | 0.00019 | 1 |
| rs17146081 | 7 | 73598918 | C | G | 0.825 | -0.034 | 0.0095 | 0.00034 | 1 |
| rs17146114 | 7 | 73640191 | A | G | 0.864 | -0.03 | 0.01 | 0.0025 | 1 |
| rs941298 | 7 | 73125263 | G | A | 0.908 | -0.014 | 0.0052 | 0.0058 | 1 |
| rs941299 | 7 | 73125179 | T | C | 0.908 | -0.014 | 0.0051 | 0.0065 | 1 |
| rs11983880 | 7 | 73122704 | C | T | 0.894 | 0.034 | 0.014 | 0.014 | 1 |
| rs941296 | 7 | 73065692 | T | C | 0.082 | -0.017 | 0.0069 | 0.015 | 1 |
| rs4717099 | 7 | 73077609 | A | G | 0.078 | -0.017 | 0.0069 | 0.018 | 1 |

(a) **rs2279463**


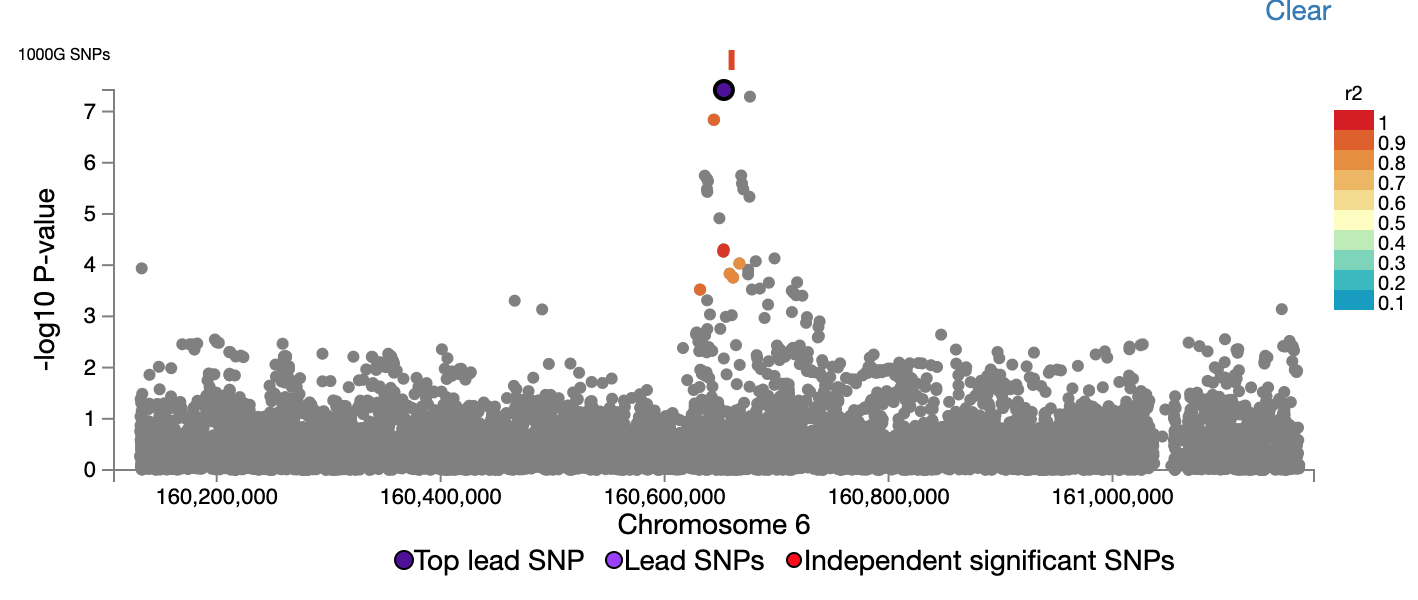


(b) rs13230509


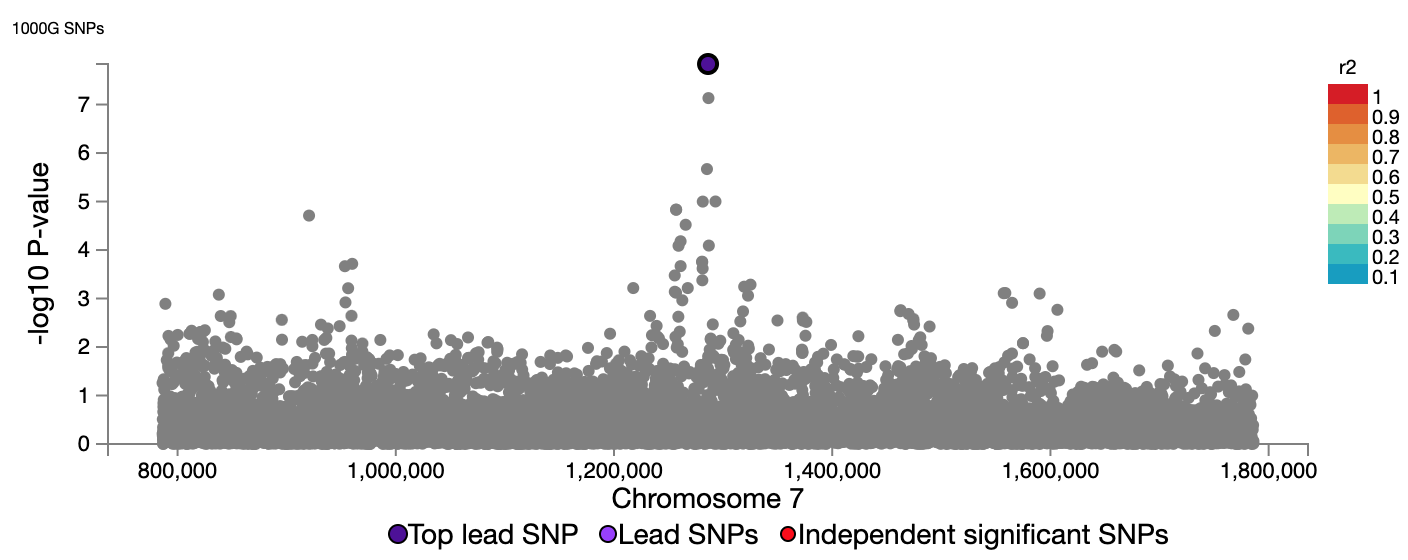


(c) rs77408001


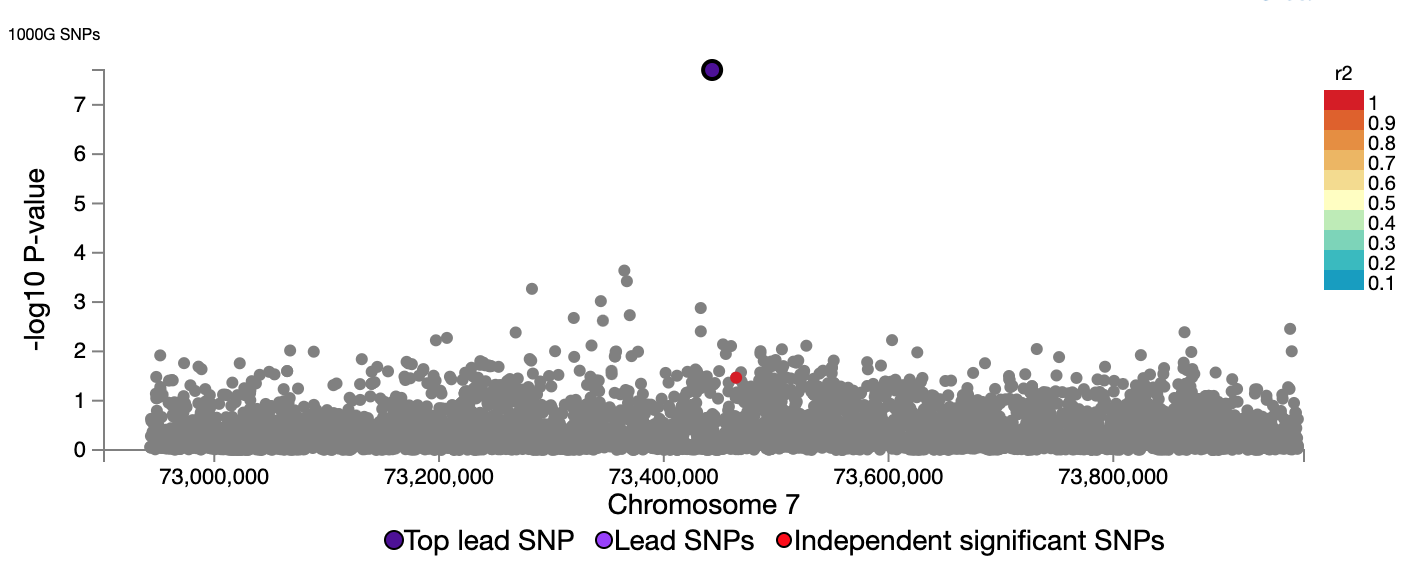


(d) rs77121243


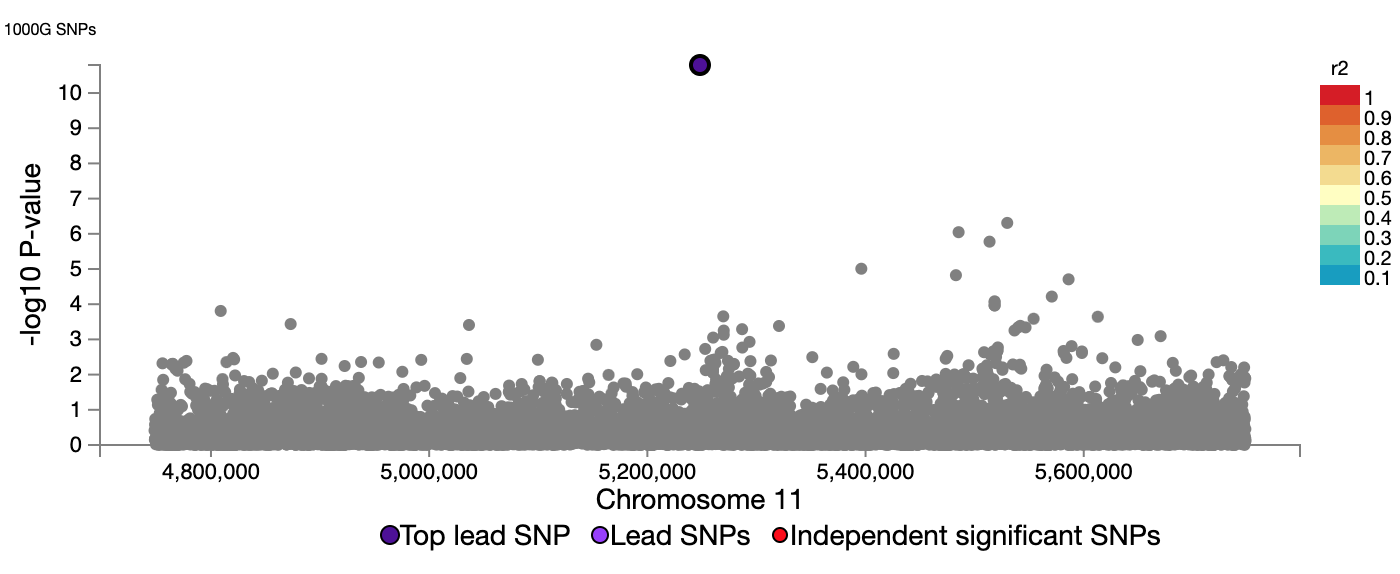


(e) rs200950799


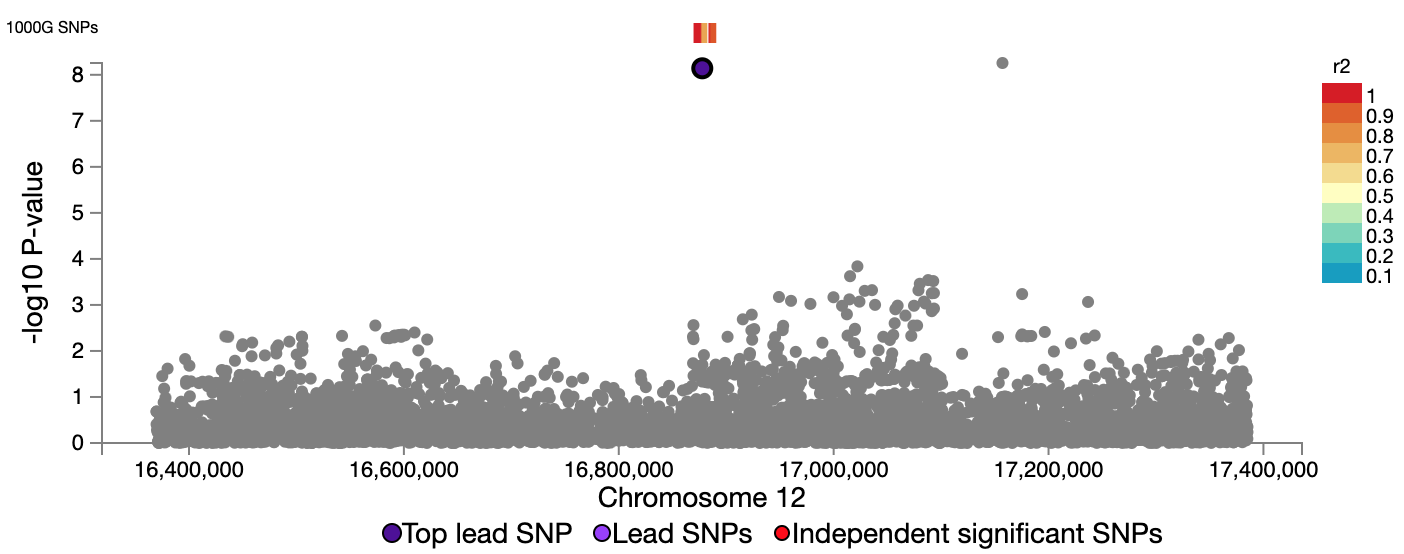


(f) rs201602445


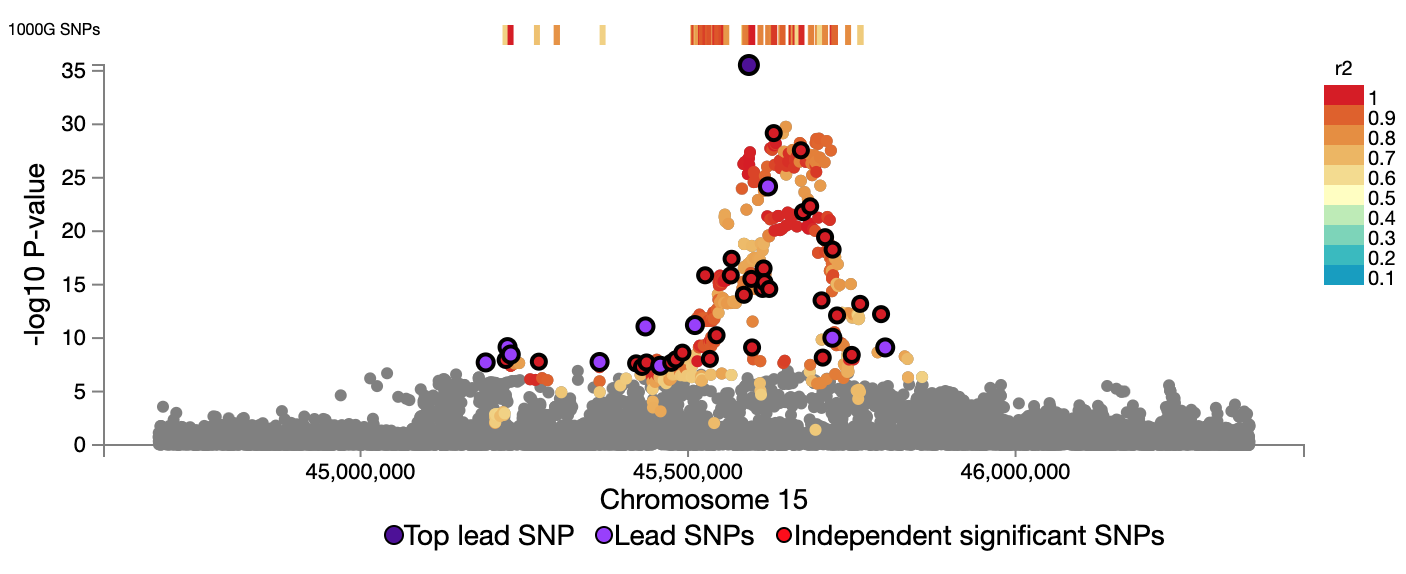


(g) rs9908131


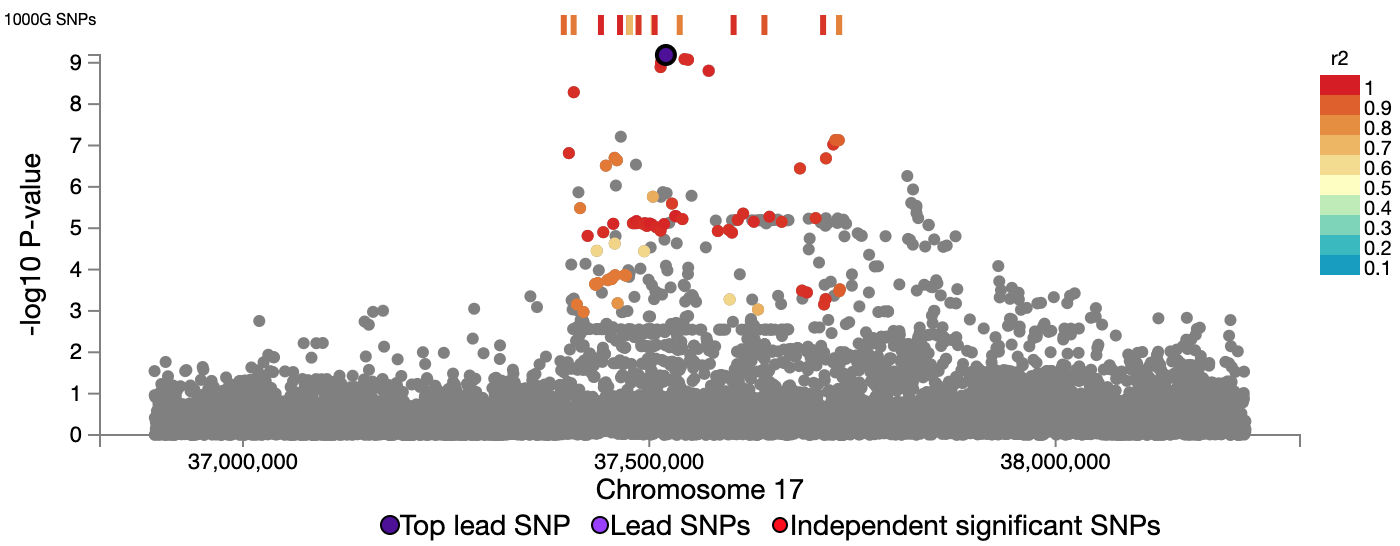


(h) rs10084572


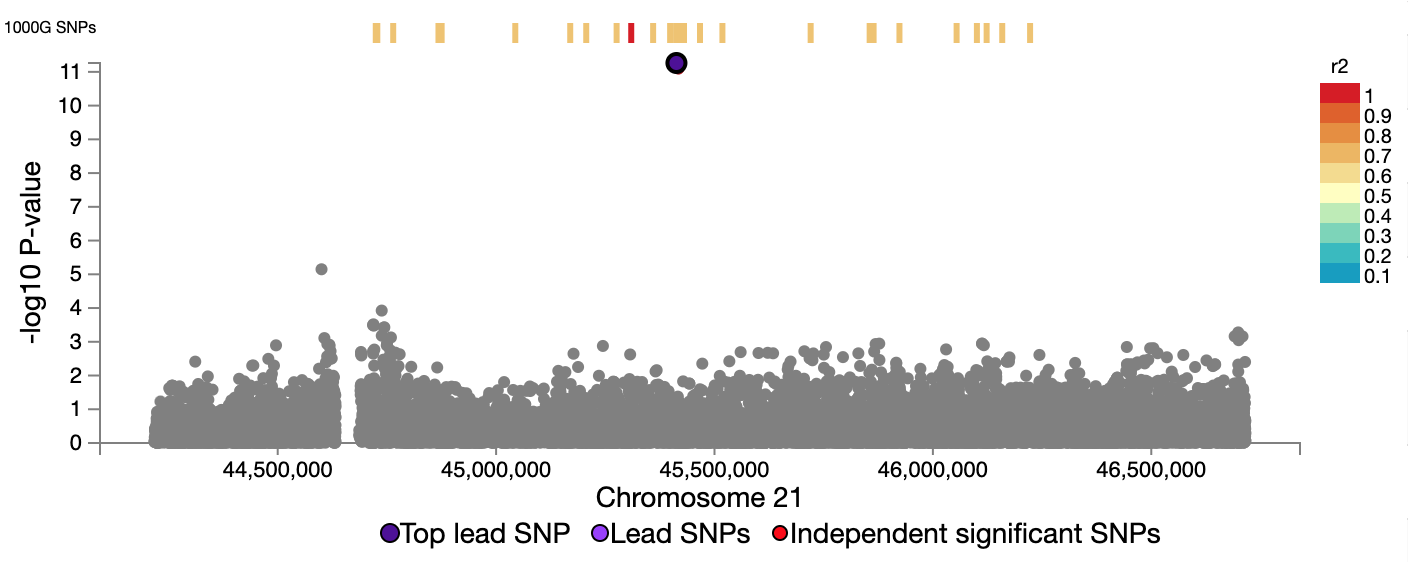


**Figure S1:** Regional association plots showing Genetic architecture of the genome-wide significant susceptibility variants for CKD (a)-(h). The most significant SNP in each region is plotted in blue. LD based on the 1000G sample is color-coded red (r2 to top SNP 0.8–1.0), orange (0.5–0.8), yellow (0.2–0.5) and blue (<0.2).

**
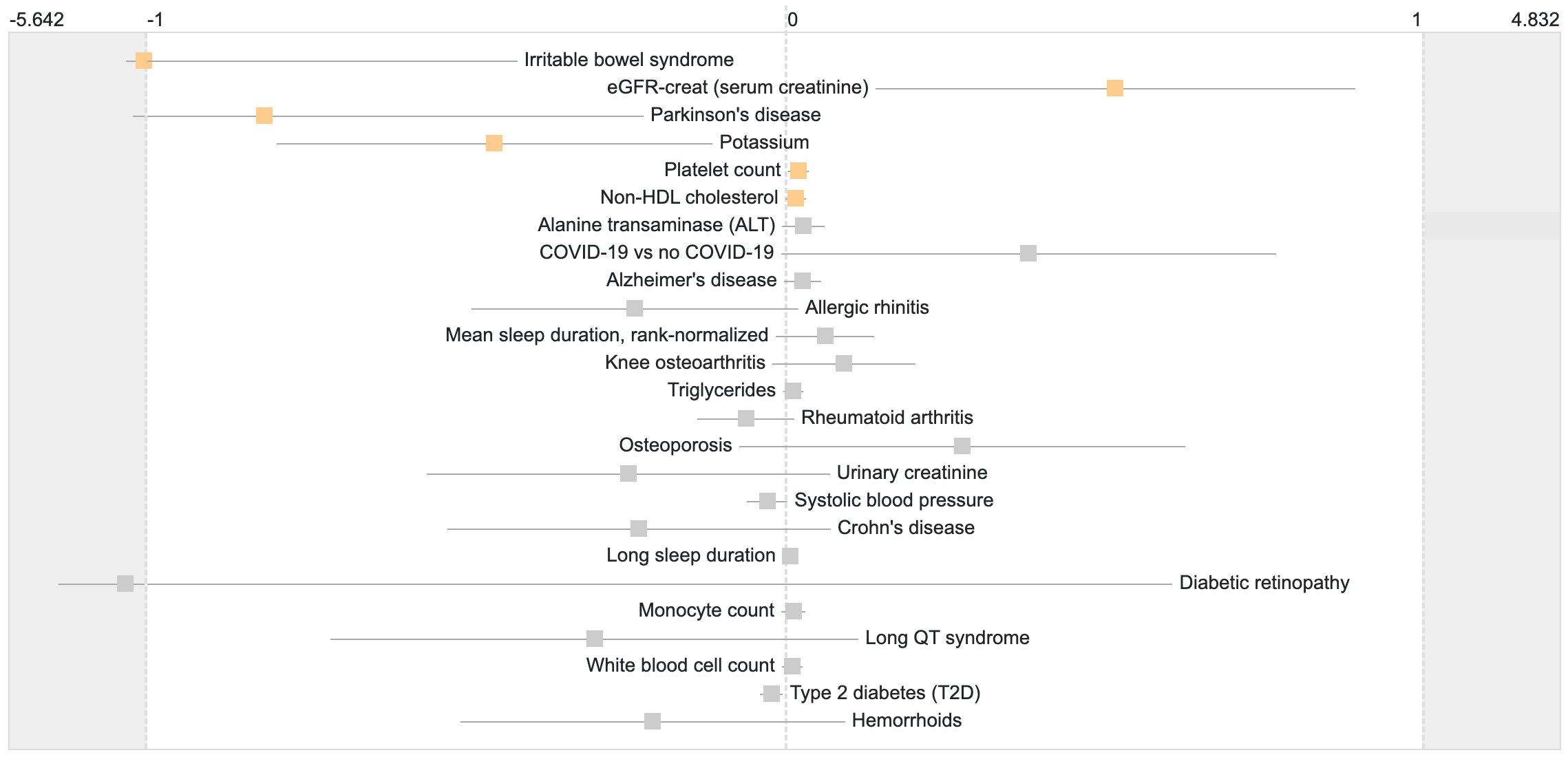
**

**Figure S2:** The Phenome-wide association (PheWAS) plot shows the significant (p ≤ 0.05) associations of 7:73443012:C:A / rs77408001 for all available traits, generated by bottom-line integrative analysis across all datasets in the Portal. The triangle data points indicate direction of effect.

**
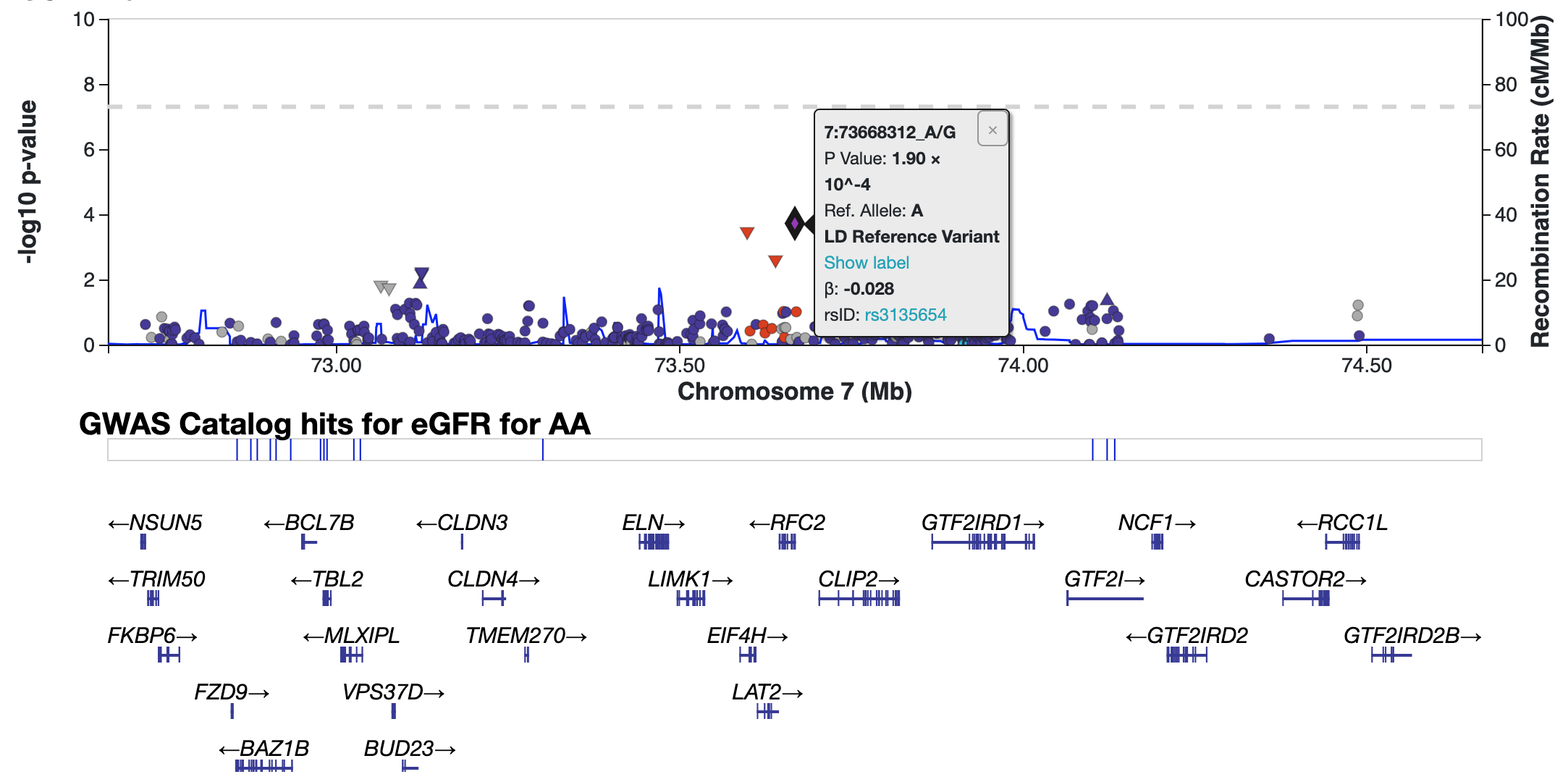
**

**Supplementary figure s3: Regional association plot for proxy SNP rs3135654 in 16474 African-ancestry individuals from the CKDGen consortium dataset at the ELN locus.**

**References**

1. Manichaikul, A., et al., *Robust relationship inference in genome-wide association studies.* Bioinformatics, 2010. **26**(22): p. 2867-73.

2. Das, S., et al., *Next-generation genotype imputation service and methods.* Nat Genet, 2016. **48**(10): p. 1284-1287.

3. Willer, C.J., Y. Li, and G.R. Abecasis, *METAL: fast and efficient meta-analysis of genomewide association scans.* Bioinformatics, 2010. **26**(17): p. 2190-1.

4. Hellwege, J.N., et al., *Mapping eGFR loci to the renal transcriptome and phenome in the VA Million Veteran Program.* Nat Commun, 2019. **10**(1): p. 3842.

5. Pattaro, C., et al., *Genetic associations at 53 loci highlight cell types and biological pathways relevant for kidney function.* Nat Commun, 2016. **7**: p. 10023.

6. Allen, N., et al., *UK Biobank: Current status and what it means for epidemiology.* Health Policy and Technology, 2012. **1**(3): p. 123-126.

7. Elliott, P., T.C. Peakman, and U.K. Biobank, *The UK Biobank sample handling and storage protocol for the collection, processing and archiving of human blood and urine.* Int J Epidemiol, 2008. **37**(2): p. 234-44.

8. Lees, J.S., et al., *Glomerular filtration rate by differing measures, albuminuria and prediction of cardiovascular disease, mortality and end-stage kidney disease.* Nat Med, 2019. **25**(11): p. 1753-1760.

9. Fatumo, S., et al., *Discovery and fine-mapping of kidney function loci in first genome-wide association study in Africans.* Hum Mol Genet, 2021. **30**(16): p. 1559-1568.

10. Mayanja, R.a.M., Tafadzwa and Soremekun, Opeyemi and Kamiza, Abram and Kintu, Christopher and Kalungi, Allan and Kalyesubula, Robert and Sande, Obondo J. and Jjingo, Daudi and Fabian, June and Robinson-Cohen, Cassianne and Franceschini, Nora and Nitsch, Dorothea and Nyrenda, Moffat and Zeggini, Eleftheria and Morris, Andrew P. and Chikowore, Tinashe and Fatumo, Segun, First GWAS of Cystatin-C Kidney Function in Continental Africa Identifies Novel Loci & Refines Known Associations, *First GWAS of Cystatin-C Kidney Function in Continental Africa Identifies Novel Loci & Refines Known Associations.* 2023.
